# Supplementary material for: Generation of a mutator parasite to drive resistome discovery in Plasmodium falciparum
Source: Nat Commun. 2023 May 27;14:3059. doi: 10.1038/s41467-023-38774-1 (PMC10224993; doi:10.1038/s41467-023-38774-1)
Supplement: Supplementary file 1 — Supplementary Information [file 41467_2023_38774_MOESM1_ESM.pdf]

Supplementary Information

Generation of a mutator parasite to drive resistome discovery in *Plasmodium falciparum*

Krittikorn Kümpornsin, Theerarat Kochakarn, Tomas Yeo, John Okombo, Madeline R Luth, Johanna Hoshizaki, Mukul Rawat, Richard D Pearson, Kyra A Schindler, Sachel Mok, Heekuk Park, Anne-Catrin Uhlemann, Gouranga P. Jana, Bikash C. Maity, Benoît Laleu, Elodie Chenu, James Duffy, Sonia Moliner Cubel, Virginia Franco, Maria G Gomez-Lorenzo, Francisco Javier Gamo, Elizabeth A Winzeler, David A Fidock, Thanat Chookajorn, Marcus CS Lee

1. Supplementary Methods
2. Supplementary Figures
3. Supplementary Tables

1. Supplementary Methods

The following compounds were synthesised.

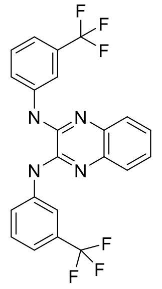

Target-MMV665794
MMV665794-08

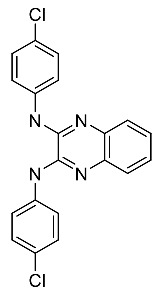

MMV665794_Target-4
MMV1582462-01

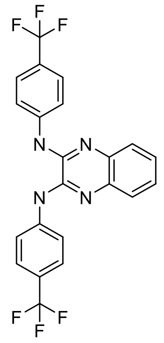

MMV665794_Target-12
MMV1582470-01

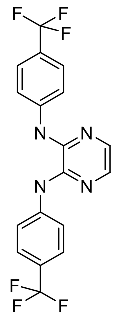

MMV665794_Set-2_Target-1
MMV1634608-01

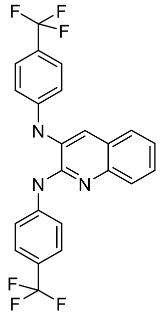

MMV665794_Set-3_Target-9
MMV1634609-01

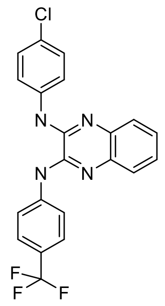

MMV665794_Set-2_Target-3
MMV1782345-01

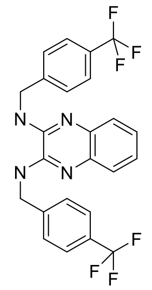

MMV665794_Set-3_Target-12
MMV1782343-01

The following compounds were purchased. MMV1582455 from Princeton BioMolecular Research, LQZ-7I, BQR695 and KDU691 from Cambridge Bioscience, STK389043, STK996213 and STL326058 from MolPort. Structures of these compounds are shown in Supplementary Figure 9.

General procedures for compound synthesis

All the starting materials and solvents were procured from commercial sources or synthesized according to the literature procedure. Organic solutions were dried over anhydrous sodium sulfate.

1H NMR Spectroscopy

1H NMR was recorded at 400.20 & 400.10, 400.17 respectively, on a Bruker Avance II & III spectrometer, using solvents from Merck Laboratories. Chemical shifts (δ, ppm) are reported

relative to the solvent peak (CDCl3: 7.25 [1H], DMSO-d6: 2.50 [1H]. Proton resonances are annotated as: chemical shift (δ), multiplicity (s, singlet; d, doublet; t, triplet; q, quartet; m, multiplet; br, broad), coupling constant (J, Hz), and number of protons.

Synthesis of Target-MMV665794

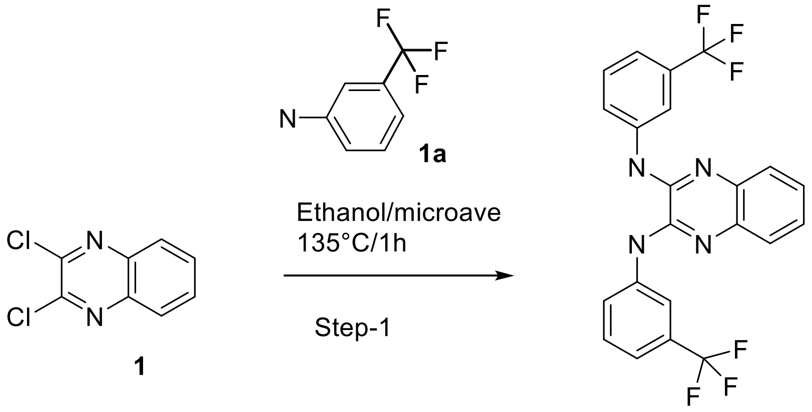

Target-MMV665794

To a solution of 2,3-dichloroquinoxaline (**1**) (100 mg, 0.503 mmol) in ethanol (5 ml) was added 3-(trifluoromethyl)aniline (**1a)** (404.52 mg, 2.51 mmol) in a microwave vial at 25°C and the resulting reaction was allowed to stir at 135°C for 1h under microwave irradiation. After consumption of starting material, reaction mixture was concentrated in vacuum to provide a crude residue. The crude residue was purified using column chromatography on silica gel using 30% CH_2Cl_2 in hexane as eluents to afford N_2,N_3-bis[3-(trifluoromethyl)phenyl]quinoxaline-2,3-diamine **(Target-MMV665794)** (35 mg, 16%) as a white solid.

Synthesis of MMV665794_Target-4 (MMV1582462-01)

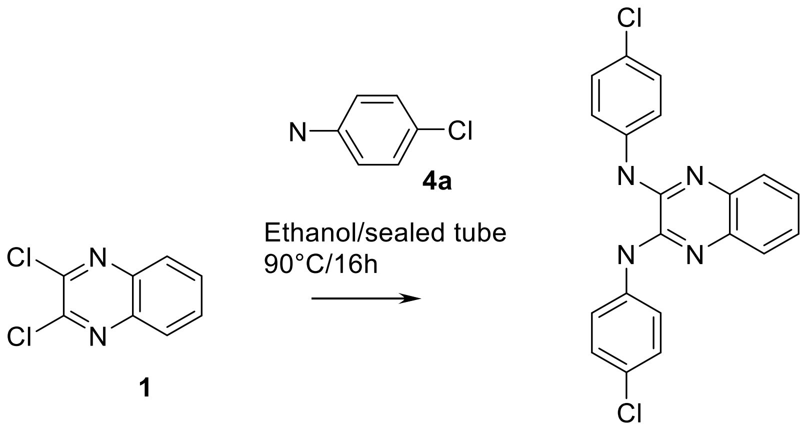

MMV665794_Target-4

MMV1582462-01

To a stirred solution of 2,3-dichloroquinoxaline (**1**) (100 mg, 0.502 mmol) in ethanol (5 ml) was added 4-chloroaniline (**4a)** (330 mg, 2.58 mmol) in a sealed tube at 25°C and the resulting reaction was allowed to stir at 90°C for 16h. After consumption of starting material (monitor by TLC), mixture was concentrated in vacuum to provide a crude residue. The crude residue was purified using column chromatography on silica gel using 3% MeOH in CH_2Cl_2 as eluents to get afford N_2,N_3-bis(4-chlorophenyl)quinoxaline-2,3-diamine (**MMV665794_Target-4)** (62 mg, 31%) as a white solid.

Synthesis of MMV665794_Target-12 (MMV1582470-01)

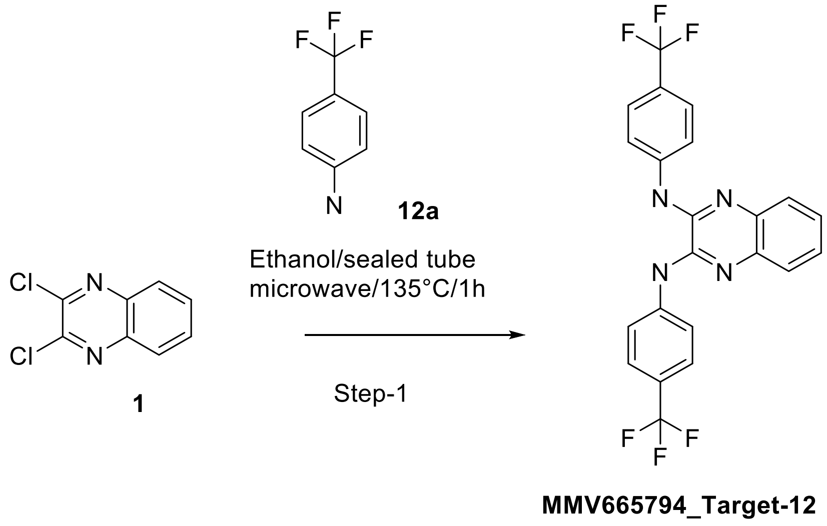

MMV665794_Target-12

MMV1582470-01

To a stirred solution of 2,3-dichloroquinoxaline (**1**) (199 mg, 1.00 mmol) in ethanol (5 ml) was added 4-(trifluoromethyl)aniline (**12a**) (805 mg, 5.0 mmol) in a microwave vial at 25°C and the resulting reaction was allowed to stir at 135°C for 1h under microwave irradiation. After consumption of starting material (monitor by TLC), reaction mixture was concentrated in vacuum to provide a crude residue. The crude residue was purified using column chromatography on silica gel using 30% CH_2Cl_2 in hexane as eluents to afford N_2,N_3-bis(4-(trifluoromethyl)phenyl)quinoxaline-2,3-diamine (**MMV665794_Target-12)** (30 mg, 7%) as a white solid.

Synthesis of MMV665794_Set-2_Target-1 (MMV1634608-01) and Set-3_Target-9 (MMV1634609-01)

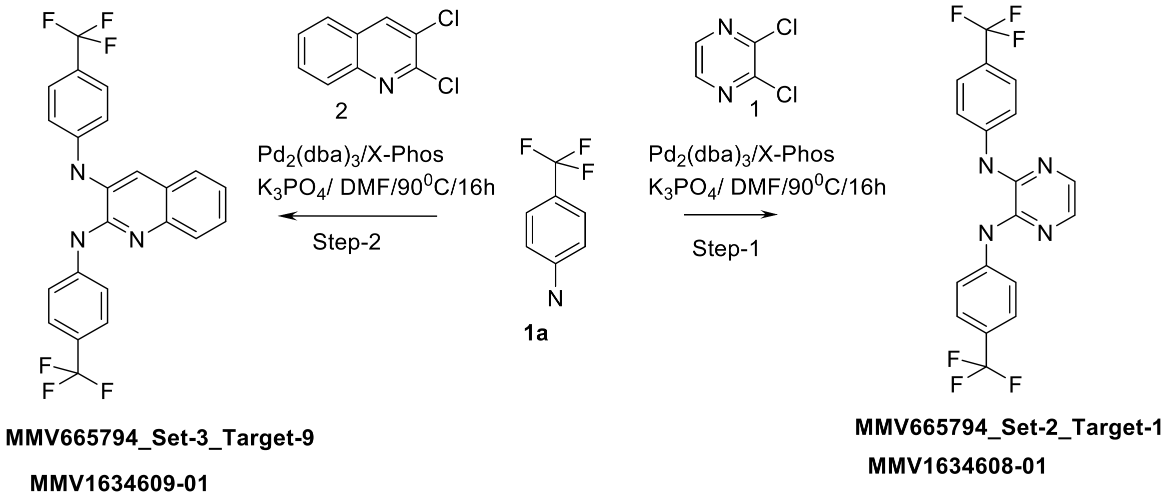

Step-1

Synthesis of compound MMV665794_Set-2_Target-1 (MMV1634608-01)

To a stirred solution of 2,3-dichloropyrazine (**1**) (300 mg, 2.014 mmol) and 4-(trifluoromethyl)aniline **(1a)** (584 mg, 3.625 mmol) in DMF (6 ml) was added K_3PO_4 (1.28 gm, 6.041 mmol) at RT. The reaction mixture was purged with argon for 20 min. Under argon atmosphere the reaction mixture was added $Pd_2(dba)_3$ (370 mg, 0.403 mmol) and X-Phos (192 mg, 0.403 mmol). Then the reaction mixture was heated to 90°C for 16h. TLC (20% EtOAc-Hexane) showed completion of the reaction. It was quenched with water and extracted with EtOAc, washed with brine, dried over Na_2SO_4 and concentrated under reduced pressure. The crude was purified by prep HPLC to afford N2,N3-bis[4-(trifluoromethyl)phenyl]pyrazine-2,3-diamine; **(MMV665794_Set-2_Target-1)** (30 mg, 5%) as pale-yellow solid.

Step-2

Synthesis of compound MMV665794_Set-3_Target-9 (MMV1634609-01)

To a stirred solution of 4-(trifluoromethyl)aniline **(1a)** (100 mg, 0.505 mmol) and 2,3-dichloroquinoline (**2)** (244 mg, 1.514 mmol) in DMF (2 ml) was added K_3PO_4 (321.35 mg, 1.514 mmol) at RT under argon atmosphere. To this reaction mixture was added $Pd_2(dba)_3$ (92 mg , 0.101 mmol) and X-Phos (48 mg, 0.101 mmol). Then the reaction mixture was heated to 90°C for 16h. TLC (20% EtOAc-Hexane) showed completion of the reaction. It was quenched with ice cold water and extracted with EtOAc, washed with brine, dried over Na_2SO_4 and concentrated under reduced pressure. The crude was purified by prep HPLC to afford N2,N3-bis[4-(trifluoromethyl)phenyl]quinoline-2,3-diamine; **MMV665794_Set-3_Target-9** (25 mg, 11%) as white solid.

Syntheses of MMV665794_Set-2_Target-3 (MMV1782345-01) and Set-3_Target-12 (MMV1782343-01)

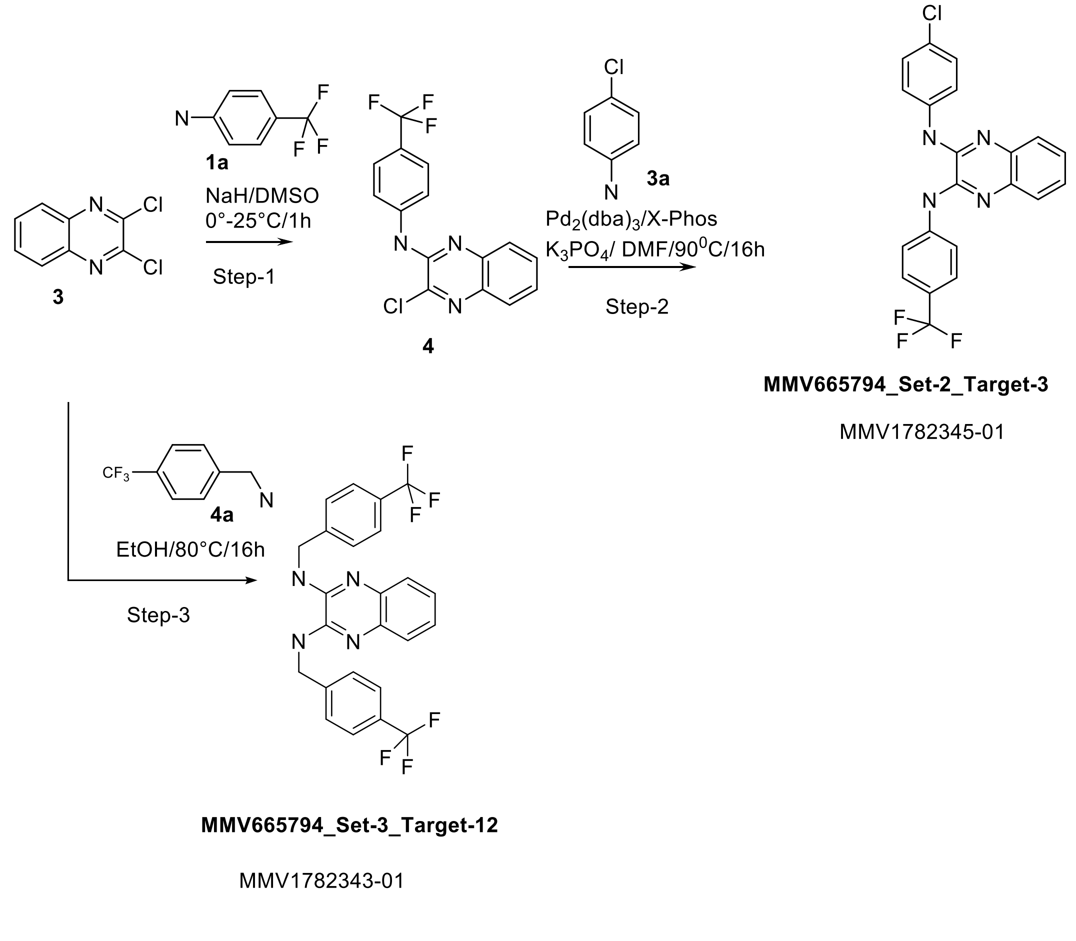

Step-1

Synthesis of compound 4

To a stirred solution of 4-(trifluoromethyl)aniline **(1a)** (485 mg, 3.015 mmol) in DMSO (10 ml) was added 60% NaH (300 mg, 7.538 mmol) at 0°C and was stirred for 10 min. Then 2,3-dichloroquinoxaline **(3)** (600 mg, 3.02 mmol) was added to this reaction mixture and allowed the reaction to stir for 1h at rt. TLC (20% EtOAc-Hexane) showed completion of the reaction. Added cold water to quench the reaction and extracted with EtOAc (25 ml x 2), washed with brine, dried over Na_2SO_4 and concentrated under reduced pressure. The crude was purified by column chromatography (8% EtOAc- Hexane) to afford 3-chloro-N-(4-(trifluoromethyl)phenyl)quinoxalin-2-amine (**4)** (510 mg, 52%) as pale yellow solid.

Step-2

Synthesis of compound MMV665794_Set-2_Target-3 (MMV1782345-01)

To a stirred solution of 3-chloro-N-(4-(trifluoromethyl)phenyl)quinoxalin-2-amine (**4)** (200 mg, 0.618 mmol) and 4-chloroaniline (**3a)** (87 mg, 0.68 mmol) in DMF (5 ml) was added Potassium phosphate (393 mg, 1.854 mmol) at room temperature under argon atmosphere. Then Tris(dibenzylideneacetone)dipalladium (0.113 g, 0.124 mmol) and Dicyclohexyl[2′,4′,6′-tris(propan-2-yl)[1,1′-biphenyl]-2-yl]phosphane (0.059 mg, 0.124 mmol) was added to this reaction mixture. Then the reaction mixture was heated to 90°C for 16h. TLC (20% EtOAc-Hexane) showed completion of the reaction. It was quenched with water and extracted with EtOAc, washed with brine, dried over Na_2SO_4 and concentrated under reduced pressure. The crude was purified by prep HPLC to afford N_2-(4-chlorophenyl)-N_3-(4-(trifluoromethyl)phenyl)quinoxaline-2,3-diamine (**MMV665794_Set-2_Target-3)** (40 mg, 16%) as pale yellow solid.

Step-3

Synthesis of compound MMV665794_Set-3_Target-12 (MMV1782343-01)**:** Ref- CR302-12794-43-F

To a stirred solution of 2,3-dichloroquinoxaline (**3)** (0.600 g, 3.015 mmol) and (4-(trifluoromethyl)phenyl)methanamine (**4a)** (1.58 gm, 9.044 mmol) in ethanol (10 ml) was heated to 80°C in a sealed tube for 16h. After completion of reaction it was concentrated, diluted with 10% MeOH-DCM, washed with water, dried over Na_2SO_4 and concentrated under reduced pressure to obtain crude. The crude was purified by column chromatography (10% EtOAc-Hexane) to afford N_2,N_3-bis(4-(trifluoromethyl)benzyl)quinoxaline-2,3-diamine (**MMV665794_Set-3_Target-12)** (22 mg, 2 %) as off white solid.

2. Supplementary Figures

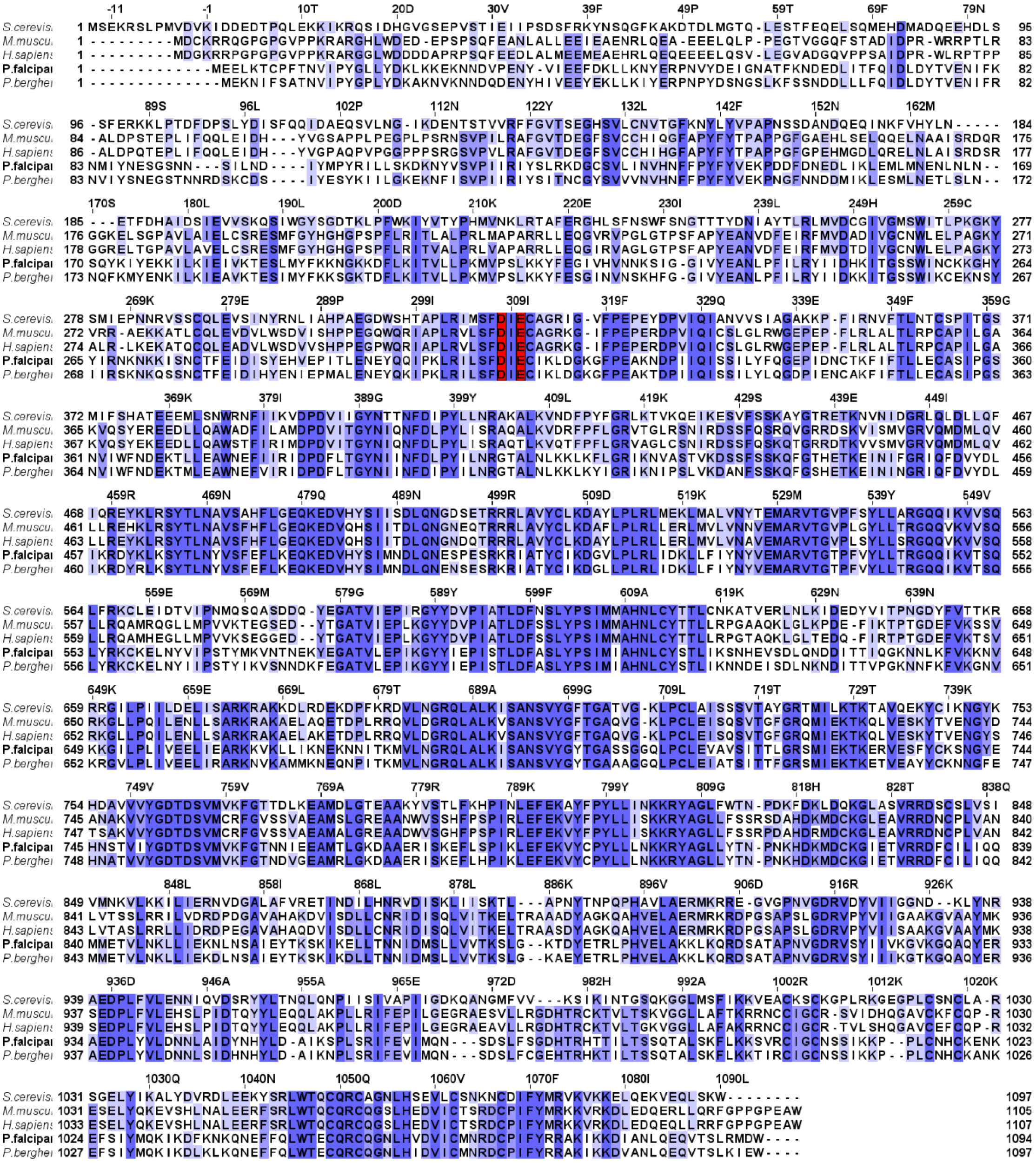

Supplementary Figure 1. Alignment of DNA polymerase δ from different species. The DNA polymerase δ catalytic subunit for yeast (*S. cerevisiae*), mouse (*M. musculus*), human (*H. sapiens*) and two malaria parasites (*P. falciparum* and *P. berghei*) were aligned using Clustal Omega. The two conserved catalytic residues of the 3′-5′ exonuclease subunit mutated in the Dd2-Polδ line (D308A / E310A) are highlighted in red.

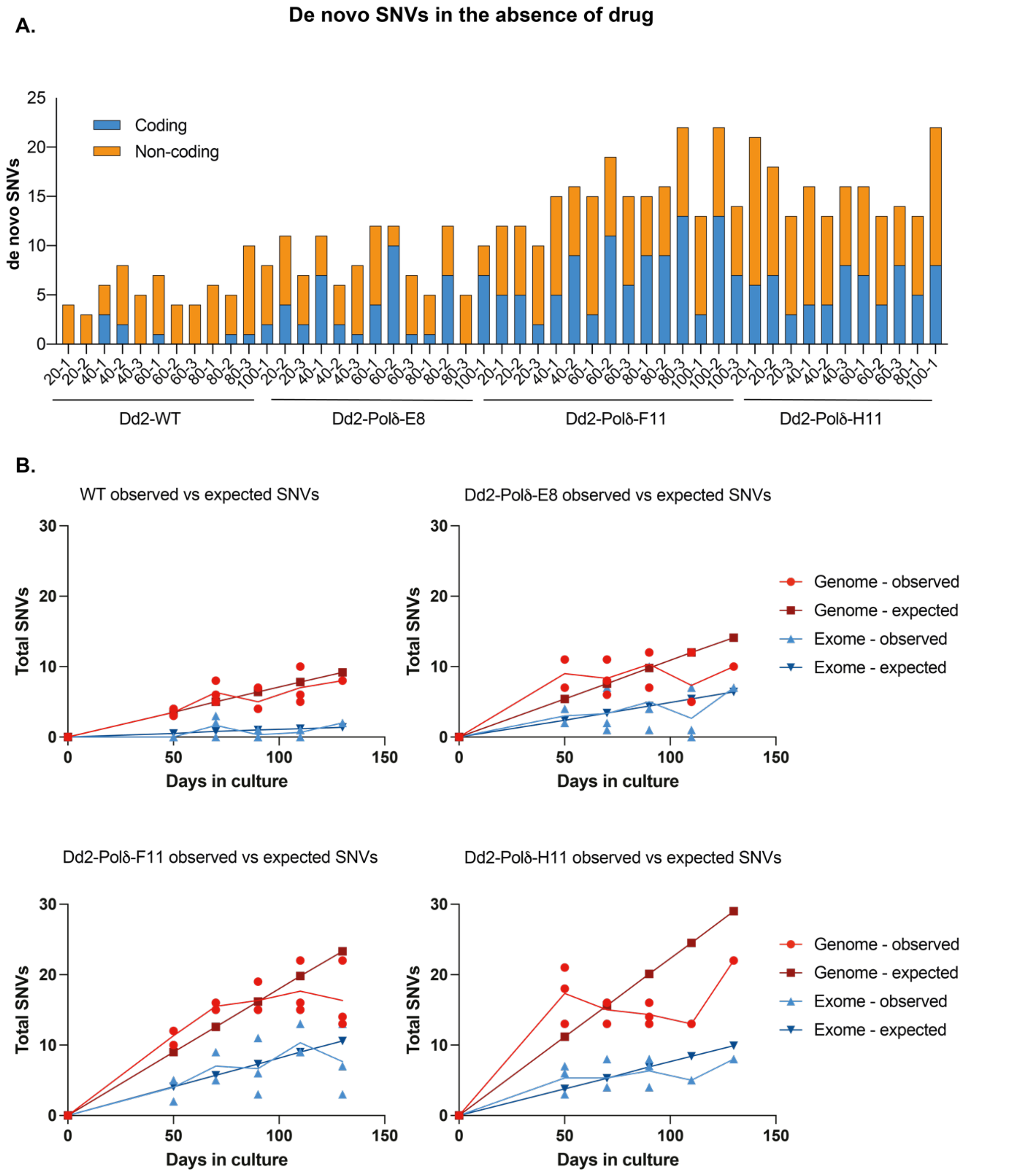

Supplementary Figure 2. ***De novo*** **SNVs observed in the absence of drug pressure. A**) SNVs observed during the mutation accumulation assay performed with Dd2-WT and three different clones of Dd2-Polδ. Parasite lines were grown in continuous culture for 100 days and sampled for whole-genome sequencing (relates to Figure 2). Labels refer to the collection day and clone number (e.g. 20-1: day 20 clone 1). **B**) SNVs observed over the assay period, plotted alongside the expected number of SNVs based on calculated mutation rate (Table 1). SNVs for Dd2-WT (n=12) and Dd2-Polδ clone E8 (n=12), F11 (n=14) and H11 (n=11) are shown. Source data are provided as a Source Data file.

A.

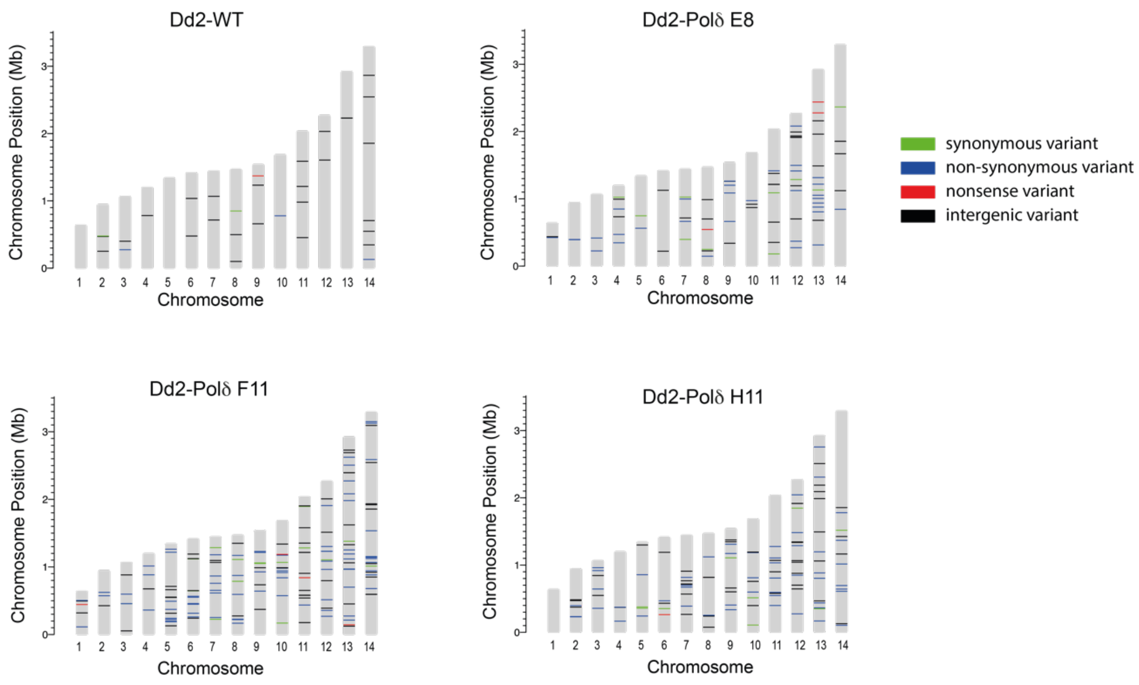

B.

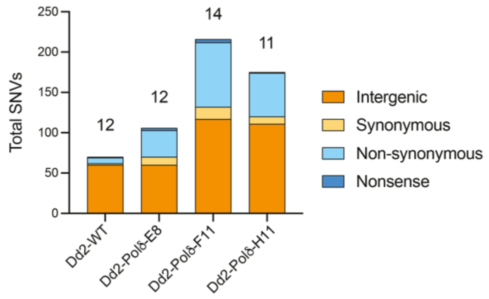

C.

Genes with different non-synonymous mutations arising in multiple lines

Gene ID	Gene Name	Lines	Mutations
PFDd2_020015000	nucleoporin NUP390, putative	PolδE8, PolδH11	E2752D, F1942L, E2030K
PFDd2_100034900	conserved Plasmodium protein, unknown function	PolδF11, PolδH11	E318*, S678L
PFDd2_120011400	DNA-directed RNA polymerase III subunit RPC2, putative	PolδE8, PolδH11	D643Y, G348C
PFDd2_130027000	protein kinase domain-containing protein, putative	PolδE8, PolδH11	Q2607K, F459L

Supplementary Figure 3. Genomic position of *de novo* SNVs. A) SNVs observed in the mutation accumulation experiment (Figure 2) are displayed for Dd2-WT and the three Dd2-Pol*δ* clones. Dashed lines indicate their position on each chromosome, and colors indicate the mutation type. **B)** Total number and type of SNVs observed across all sequence clones (number of independent clones shown above each bar). **C)** List of genes with multiple different non-synonymous mutations observed during the mutation accumulation (no drug) experiment. Source data are provided as a Source Data file.

A.

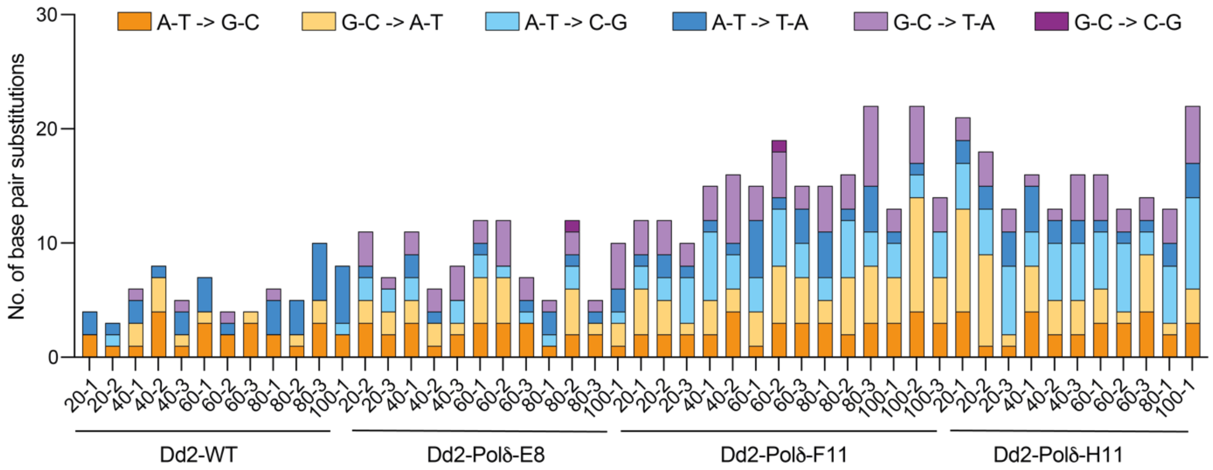

B.

		Base pair substitutions					
		Transitions		Transversions			
Parasite line	Ts:Tv	A:T > G:C	G:C > A:T	A:T > T:A	G:C > T:A	A:T > C:G	G:C > C:G
Dd2-WT	1.04	2	0.9	2.3	0.3	0.2	0
Dd2-Polδ-E8	0.90	2.2	2	1	2.3	1.3	0.08
Dd2-Polδ-F11	0.72	2.6	3.9	1.9	3.6	3.4	0.07
Dd2-Polδ-H11	0.66	2.6	3.7	2.1	2.6	4.8	0

Base-pair substitutions in Dd2-WT and Dd2-Polδ lines in drug-free media over 100 days. The number of base-pair substitutions are averaged for clones collected during day 20-100.

Supplementary Figure 4. Transition:transversion (Ts:Tv) ratio of base pair substitutions. The base pair substitutions for transition (A:T → G:C and G:C → A:T) and transversion (A:T → T:A, G:C → T:A, A:T → C:G, and G:C → C:G) were examined in the Dd2-WT and Dd2-Polδ clones. **A**) Base pair substitutions in Dd2-WT and three Dd2-Pol*δ* lines in drug-free media over 100 days. Labels refer to the collection day and clone number (e.g. 20-1: day 20 clone 1). **B**) The number of base-pair substitutions were averaged for clones of each line collected during the 100-day assay. The Ts:Tv ratio for Dd2-WT was 1.04, whereas the Ts:Tv ratio for the Dd2-Polδ clones ranged from 0.66 - 0.90. This moderately decreased Ts:Tv in Dd2-Polδ indicated that base pair substitutions tend towards transversions. This was evident especially for base-pair changes from G:C → T:A and A:T → C:G that showed an increased frequency of 7-12 fold and 5-24 fold, respectively. In addition, the transitions from G:C → A:T in Dd2-Polδ increased about 2-4 fold. Source data are provided as a Source Data file.

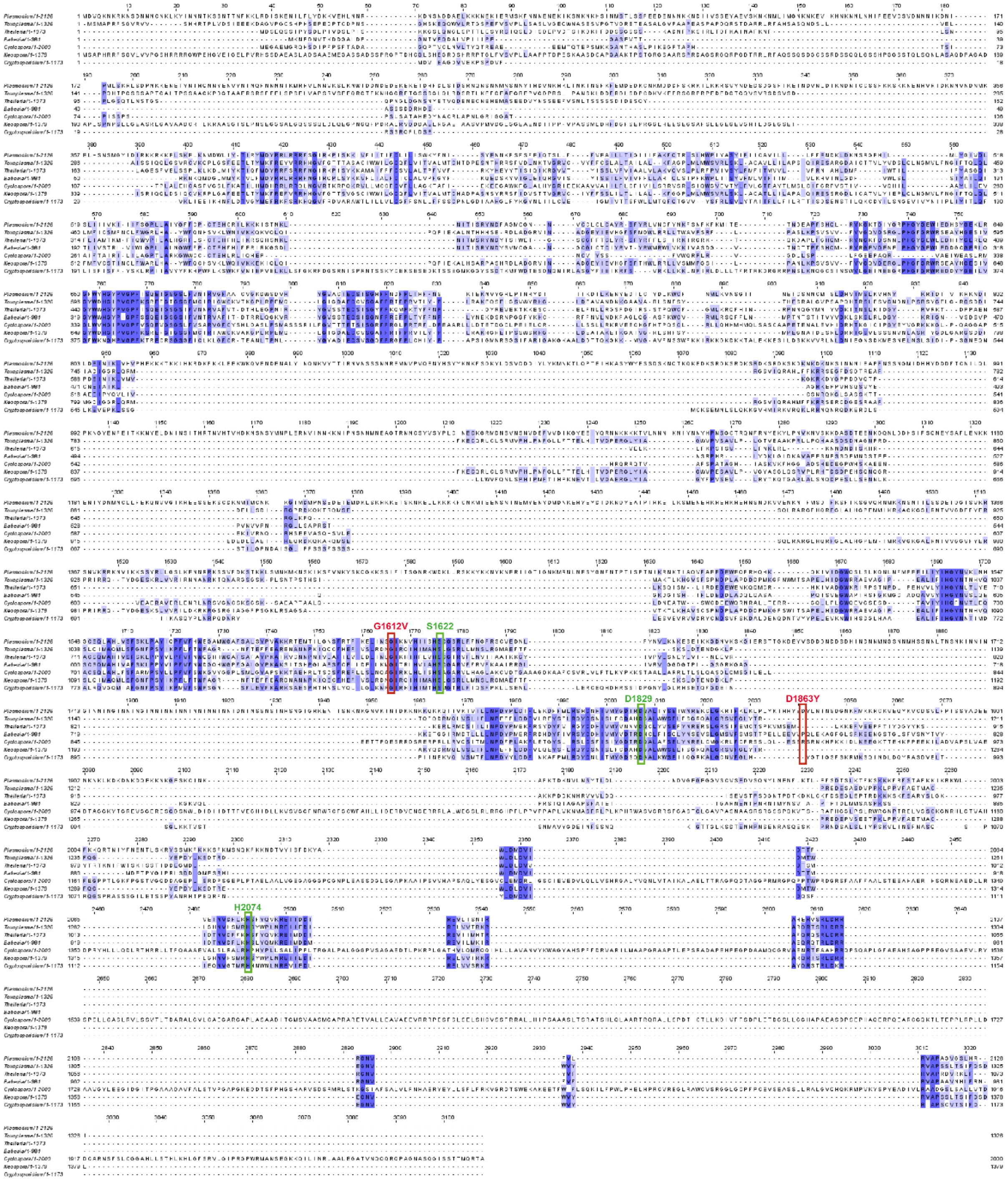

Supplementary Figure 5. Alignment of QPR1 in different apicomplexan species. Putative homologs of PfQRP1 (PF3D7_1359900) were identified using BLAST and aligned with Clustal Omega. Shown are *Toxoplasma gondii* (TGME49_289880), *Theileria parva* (TpMuguga_02g02080), *Babesia bovis* (BBOV_II004840), *Neospora caninum* (NCLIV_042110), *Cyclospora cayetanensis* (cyc_01400) and *Cryptosporidium parvum* (cgd3_590). Mutations involved in quinoxaline resistance are shown in red (G1612V & D1863Y), and a putative catalytic triad (Ser-His-Asp) is highlighted in green.

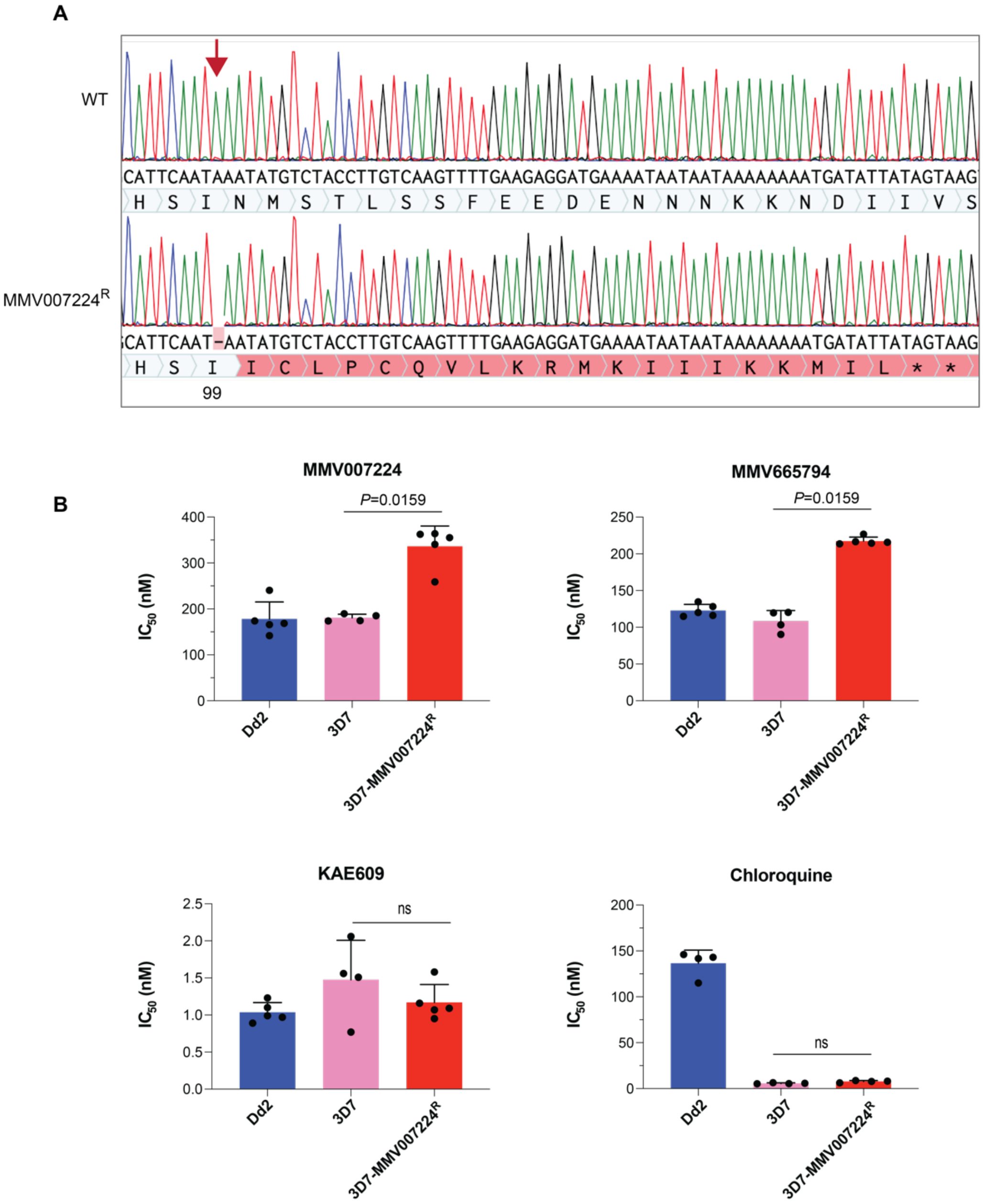

Supplementary Figure 6. MMV007224-selected parasite acquiring a frameshift in QRP1 confers low-grade resistance to its quinoxaline analogue (MMV665794). A) Sanger sequencing of the parasite line 3D7-MMV007224^{R}. The 1 bp deletion at codon Ile99 (red arrow) causes a frameshift resulting in a premature stop codon at amino acid 120 out of 2126. **B)** Drug assays showed that the MMV007224-selected line (3D7 background) shows low-level resistance to both MMV007224 and its analogue MMV665794, but not the control compounds KAE609 and chloroquine. Each dot represents a biological replicate, with mean±SD shown, and statistical significance relative to the wild type 3D7 line determined by two-sided Mann-Whitney *U* test. The following independent biological replicates were performed: MMV007224: Dd2 (5), 3D7 (4), 3D7-MMV077224R (5); MMV665794: Dd2 (5), 3D7 (4), 3D7-MMV077224R (5); KAE609: Dd2 (5), 3D7 (4), 3D7-MMV077224R (5); Chloroquine: Dd2 (4), 3D7 (4), 3D7-MMV077224R (4). Source data are provided as a Source Data file.

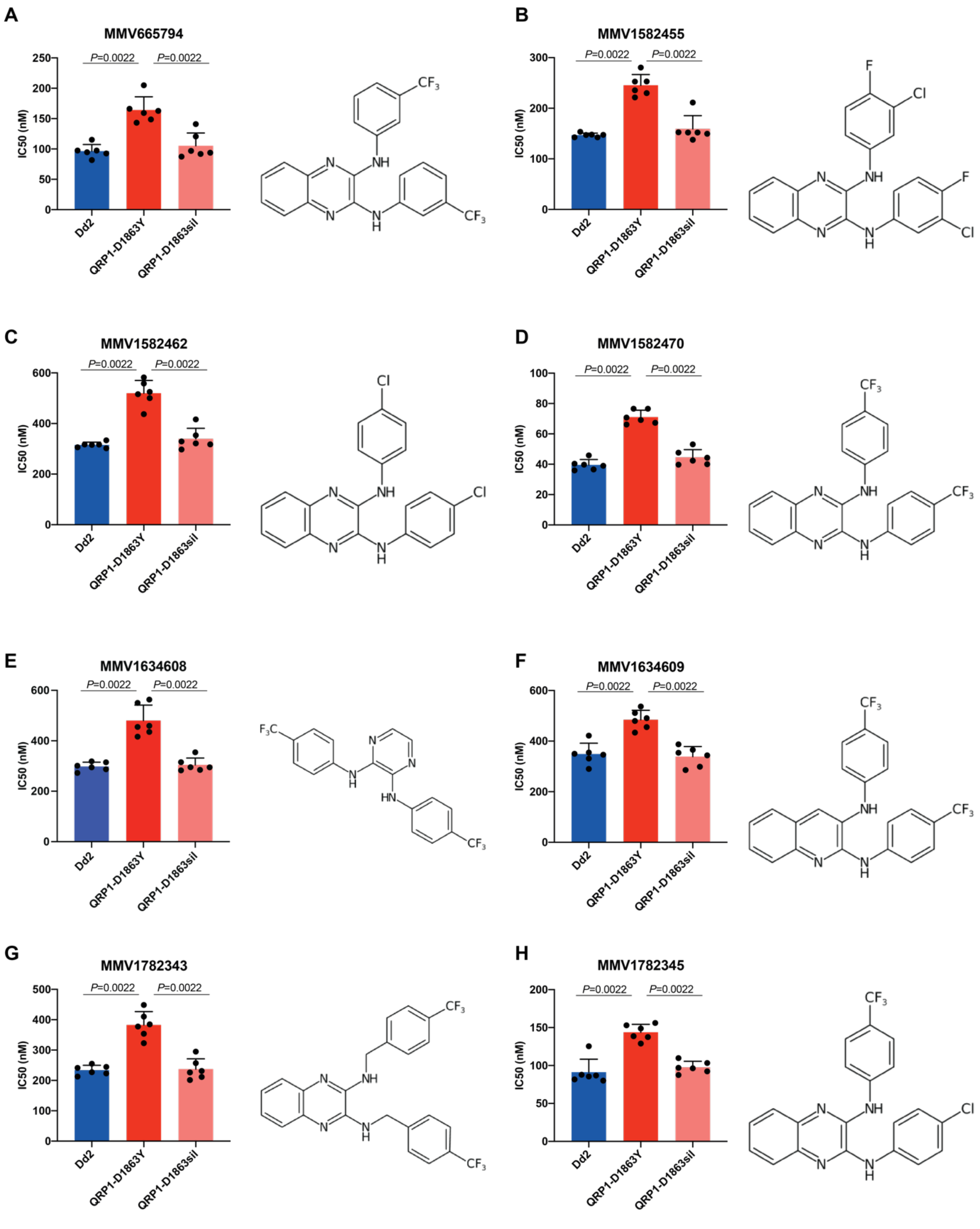

A
MMV665794
P=0.0022
P=0.0022
IC50 (nM)
250
200
150
100
50
0
Dd2
QRP1-D1863Y
QRP1-D1863sil
CF3
N
NH
N
N
H
CF3
B
MMV1582455
P=0.0022
P=0.0022
IC50 (nM)
300
200
100
0
Dd2
QRP1-D1863Y
QRP1-D1863sil
F
Cl
N
NH
N
N
H
F
Cl
C
MMV1582462
P=0.0022
P=0.0022
IC50 (nM)
600
400
200
0
Dd2
QRP1-D1863Y
QRP1-D1863sil
Cl
N
NH
N
N
H
Cl
D
MMV1582470
P=0.0022
P=0.0022
IC50 (nM)
100
80
60
40
20
0
Dd2
QRP1-D1863Y
QRP1-D1863sil
CF3
N
NH
N
N
H
CF3
E
MMV1634608
P=0.0022
P=0.0022
IC50 (nM)
600
400
200
0
Dd2
QRP1-D1863Y
QRP1-D1863sil
F3C
N
N
N
H
HN
CF3
F
MMV1634609
P=0.0022
P=0.0022
IC50 (nM)
600
400
200
0
Dd2
QRP1-D1863Y
QRP1-D1863sil
CF3
NH
N
N
H
CF3
G
MMV1782343
P=0.0022
P=0.0022
IC50 (nM)
500
400
300
200
100
0
Dd2
QRP1-D1863Y
QRP1-D1863sil
CF3
N
NH
N
N
H
CF3
H
MMV1782345
P=0.0022
P=0.0022
IC50 (nM)
200
150
100
50
0
Dd2
QRP1-D1863Y
QRP1-D1863sil
CF3
N
NH
N
N
H
Cl

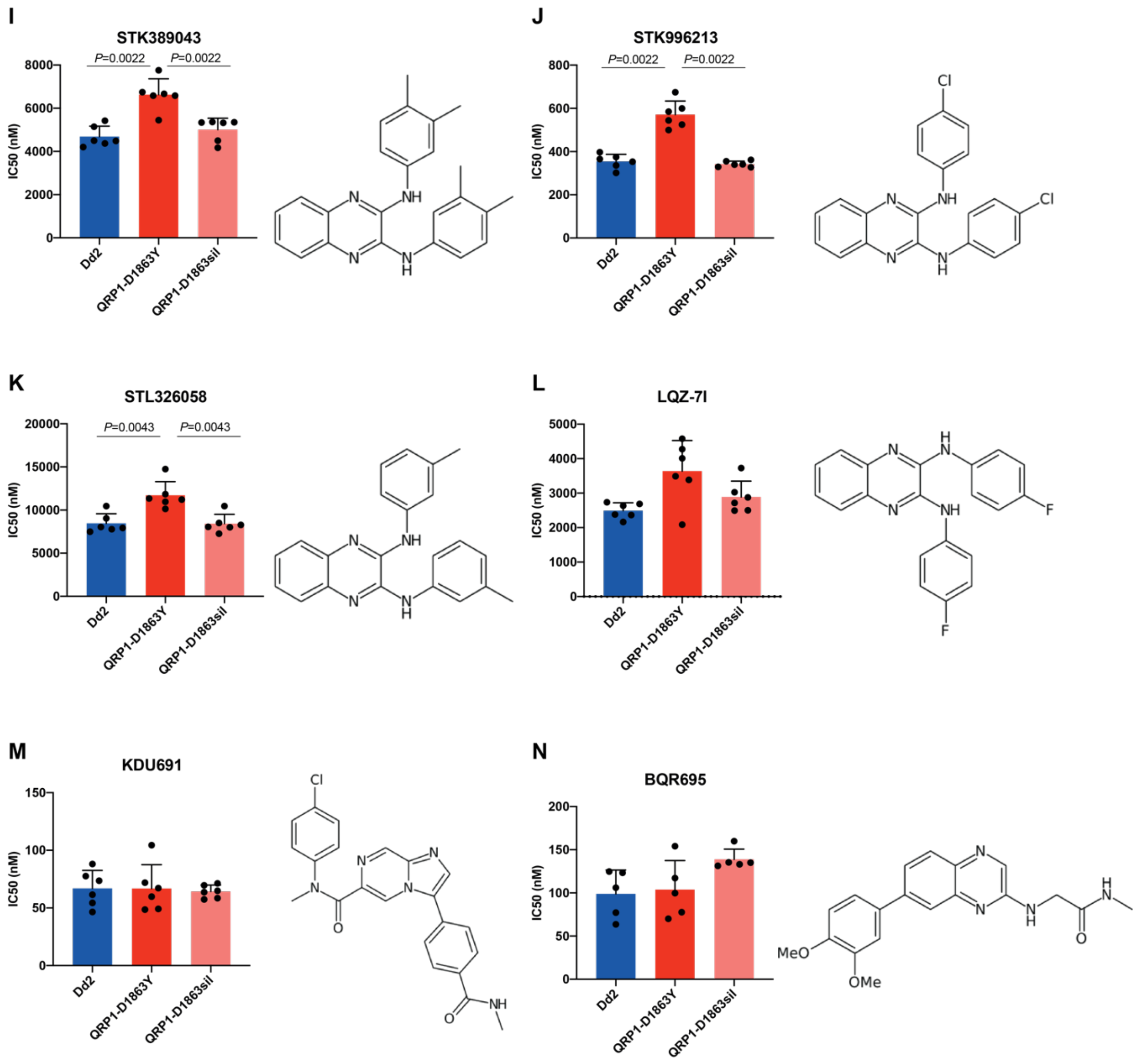

Panel	Compound	SMILES	Chemical name
A	MMV665794	FC(c1cc(Nc2c(Nc3cc(C(F)(F)F)ccc3)nc(c4n2)cccc4)ccc1)(F)F	N2,N3-bis(3-(trifluoromethyl)phenyl)quinoxaline-2,3-diamine
B	MMV1582455	N(c1ccc(F)c(Cl)c1)c2c(Nc3ccc(F)c(Cl)c3)nc4c(cccc4)n2	N,N'-bis(3-chloro-4-fluorophenyl)quinoxaline-2,3-diamine
C	MMV1582462	c1(c(ccc2)nc(Nc3ccc(Cl)cc3)c(Nc4ccc(Cl)cc4)n1)c2	N,N'-bis(4-chlorophenyl)quinoxaline-2,3-diamine
D	MMV1582470	FC(F)(F)c(cc1)ccc1Nc(nc2c(n3)cccc2)c3Nc(c4)ccc(C(F)(F)F)c4	N2,N3-bis(4-(trifluoromethyl)phenyl)quinoxaline-2,3-diamine
E	MMV1634608	c1nc(Nc2ccc(C(F)(F)F)cc2)c(Nc3ccc(C(F)(F)F)cc3)nc1	N2,N3-bis(4-(trifluoromethyl)phenyl)pyrazine-2,3-diamine
F	MMV1634609	c(cc(c1Nc2ccc(C(F)(F)F)cc2)Nc3ccc(C(F)(F)F)cc3)(cccc4)c4n1	N2,N3-bis(4-(trifluoromethyl)phenyl)quinoline-2,3-diamine
G	MMV1782343	c(c(ccc1CNc(c(NCc2ccc(C(F)(F)F)cc2)nc3c4cccc3)n4)C(F)(F)F)c1	N2,N3-bis(4-(trifluoromethyl)benzyl)quinoxaline-2,3-diamine
H	MMV1782345	c(nc1Nc2ccc(Cl)cc2)(cccc3)c3nc1Nc4ccc(C(F)(F)F)cc4	N2-(4-chlorophenyl)-N3-(4-(trifluoromethyl)phenyl)quinoxaline-2,3-diamine
I	STK389043	Cc1ccc(Nc2nc3ccccc3nc2Nc2ccc(C)c(C)c2)cc1C	N2,N3-bis(3,4-dimethylphenyl)quinoxaline-2,3-diamine
J	STK996213	Clc1ccc(Nc2nc3ccccc3nc2Nc2ccc(Cl)cc2)cc1	N2,N3-bis(4-chlorophenyl)quinoxaline-2,3-diamine
K	STL326058	Cc1cccc(Nc2nc3ccccc3nc2Nc2cccc(C)c2)c1	N2,N3-di-m-tolylquinoxaline-2,3-diamine
L	LQZ-7I	C1=CC=C2C(=C1)N=C(C(=N2)NC3=CC=C(C=C3)F)NC4=CC=C(C=C4)F	N2,N3-bis(4-fluorophenyl)quinoxaline-2,3-diamine
M	KDU691	CNC(=O)C1=CC=C(C=C1)C2=CN=C3N2C=C(N=C3)C(=O)N(C)C4=CC=C(C=C4)Cl	N-(4-chlorophenyl)-N-methyl-3-(4-(methylcarbamoyl)phenyl)imidazo[1,2-a]pyrazine-6-carboxamide
N	BQR-695	CNC(=O)CNC1=CN=C2C=CC(=CC2=N1)C3=CC(=C(C=C3)OC)OC	2-((7-(3,4-dimethoxyphenyl)quinoxalin-2-yl)amino)-N-methylacetamide

Supplementary Figure 7. Drug susceptibility of CRISPR-edited QRP1 lines against quinoxaline analogues. IC_{50} values of CRISPR-edited QRP1 lines encoding the D1863Y mutant or silent control, alongside the parental wild type Dd2, were tested against a panel of quinoxaline-like analogs synthesised (panels A-H) or sourced commercially (I-L), as well as two compounds (M-N) with an unrelated scaffold and mode-of-action. Each dot represents a biological replicate, with mean±SD shown, and statistical significance relative to wild type Dd2 or the silent-edited control line determined by two-sided Mann-Whitney *U* test. n=5 (BQR695) and n=6 (all other compounds) independent biological replicates were performed. Source data are provided as a Source Data file.

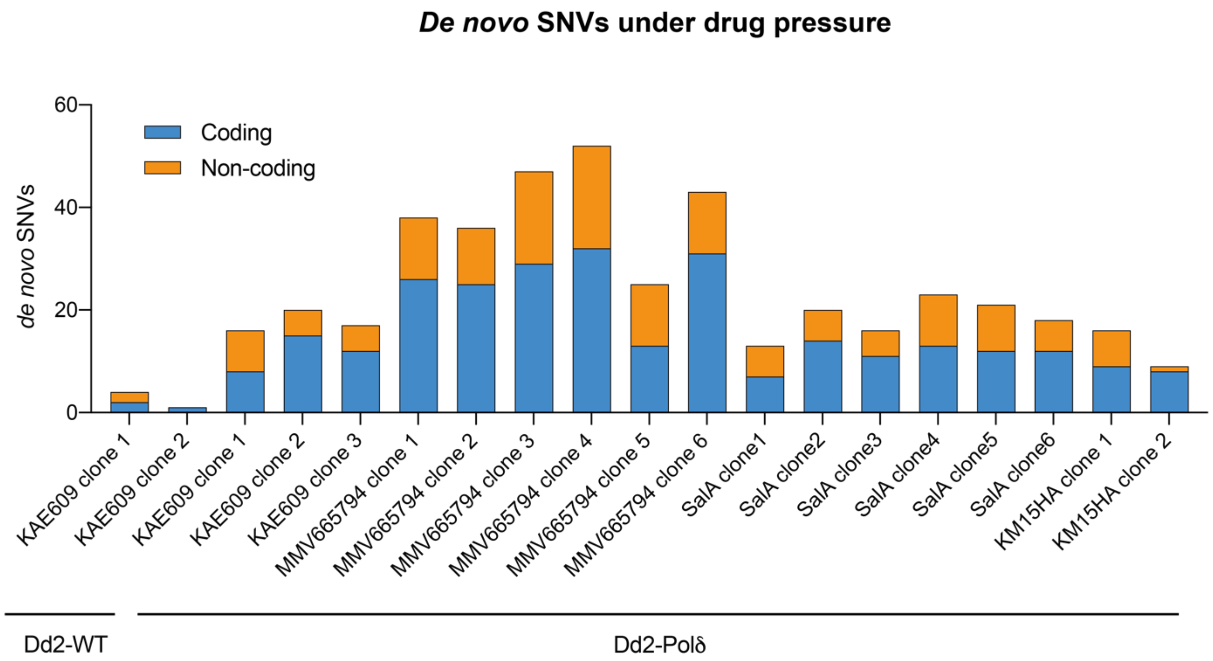

Supplementary Figure 8. *De novo* SNVs observed in the presence of drug pressure. SNVs observed in clonal drug-evolved parasites (WT or Dd2-Pol*δ* clone H11). Relates to Figure 6 and Supplementary Data 3. Source data are provided as a Source Data file.

A.

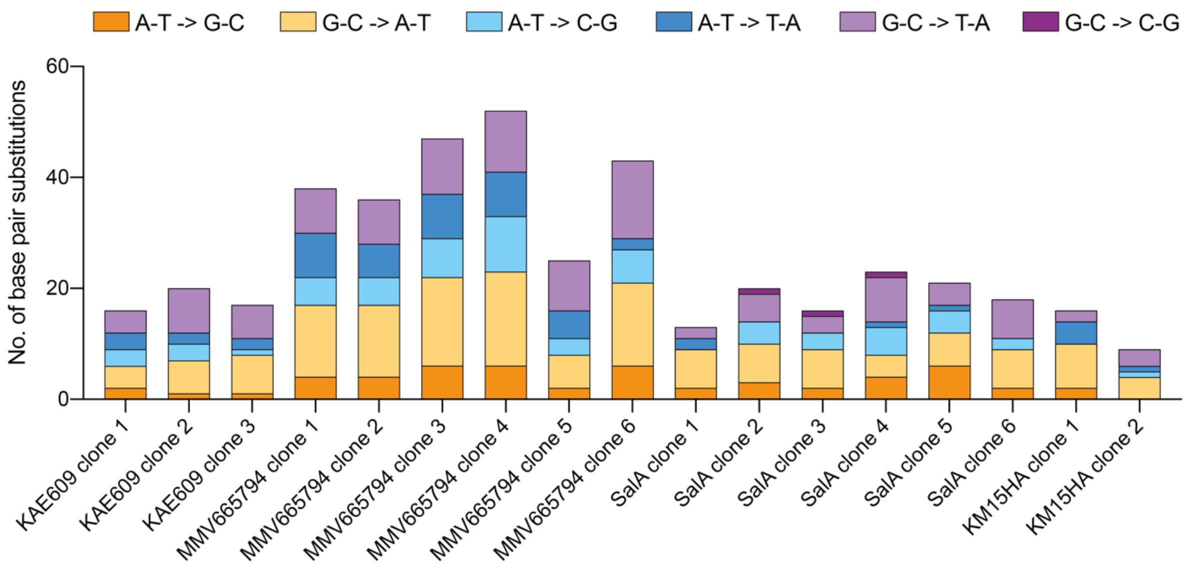

B.

		Base pair substitutions					
		Transitions		Transversions			
Compound	Ts:Tv	A:T > G:C	G:C > A:T	A:T > T:A	G:C > T:A	A:T > C:G	G:C > C:G
KAE609	0.68	1.3	5.7	2.3	6.0	2.3	0
MMV665794	0.81	4.7	13.3	6.2	10.0	6.0	0
Salinopostin A	1.05	3.2	6.3	0.7	4.8	3.0	0.5
KM15HA	1.30	1.0	6.0	2.5	2.5	0.5	0
Dd2-Polδ-H11	0.66	2.6	3.7	2.1	2.6	4.8	0

Base-pair substitutions in Dd2-Polδ under drug pressure. The number of base-pair substitutions are averaged for clones.

Supplementary Figure 9. Transition:transversion (Ts:Tv) ratios of base pair substitutions in cultures exposed to drug-pressure. A) Base pair substitutions in the Dd2-Pol*δ* clone H11 after *in vitro* evolution of resistance to KAE609, MMV665794, Salinopostin A, and KM15HA. **B**) The number of base-pair substitutions were averaged for clones of each selection. Source data are provided as a Source Data file.

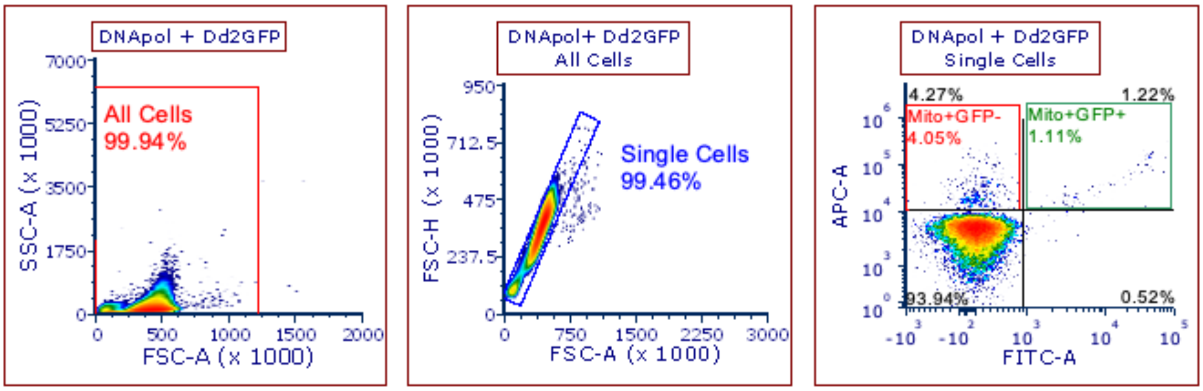

Supplementary Figure 10. Flow cytometry gating strategy. Relates to Fig. 1c. Parasites were stained with MitoTracker Deep Red FM as detailed in the Methods, and analyzed on a CytoFlex S flow cytometer. Events were gated using forward scatter area (FSC-A) vs side scatter area (SSC-A), and the population of single cells gated using forward scatter area vs height (FSC-H). Total parasite population, detected by MitoTracker Deep Red FM staining, was distinguished from uninfected RBCs (lower left) in the APC channel, with GFP-positive parasites detected in the FITC channel.

3. Supplementary Tables

Supplementary Table 1. The coordinates of the Dd2 core genome, translated from 3D7 core genome coordinates.

Chromosome	Start coordinate	Stop coordinate
PfDd2_01	49837	414782
PfDd2_01	417189	533792
PfDd2_02	947	340952
PfDd2_02	344102	755243
PfDd2_03	50904	577484
PfDd2_03	579844	981145
PfDd2_04	51063	505798
PfDd2_04	563790	590934
PfDd2_04	593460	883860
PfDd2_04	920060	1080606
PfDd2_05	41431	460387
PfDd2_05	462029	1326561
PfDd2_06	47112	454148
PfDd2_06	456464	698184
PfDd2_06	710482	1263370
PfDd2_07	53632	483475
PfDd2_07	566150	769157
PfDd2_07	771626	1339473
PfDd2_08	70570	295699
PfDd2_08	298062	424187
PfDd2_08	468417	1365003
PfDd2_09	73262	1234907
PfDd2_09	1237230	1464236
PfDd2_10	45574	1550128
PfDd2_11	20563	740907
PfDd2_11	743186	1915931
PfDd2_12	37277	742849
PfDd2_12	756229	1266945
PfDd2_12	1269177	1671085
PfDd2_12	1747106	2165416
PfDd2_13	79097	1180050
PfDd2_13	1182374	2807423
PfDd2_14	17466	1051096
PfDd2_14	1054725	3234766

Supplementary Table 2. The number of *de novo* SNVs in Dd2-WT and Dd2-Polδ occurring during mutation accumulation assay.

Parasite naming system in this table is indicated as "strain - days in culture - clone#"

Day 0 of each line was used as a baseline for counting *de novo* SNVs.

Parasite	Coding region	Non-coding region	Core genome
**Dd2-1-1**	**0**	**0**	**0**
Dd2-20-1	0	4	4
Dd2-20-2	0	3	3
Dd2-40-1	3	3	6
Dd2-40-2	2	6	8
Dd2-40-3	0	5	5
Dd2-60-1	1	6	7
Dd2-60-2	0	4	4
Dd2-60-3	0	4	4
Dd2-80-1	0	6	6
Dd2-80-2	1	4	5
Dd2-80-3	1	9	10
Dd2-100-1	2	6	8
**poldmE8-1-1**	**0**	**0**	**0**
poldmE8-20-2	4	7	11
poldmE8-20-3	2	5	7
poldmE8-40-1	7	4	11
poldmE8-40-2	2	4	6
poldmE8-40-3	1	7	8
poldmE8-60-1	4	8	12
poldmE8-60-2	10	2	12
poldmE8-60-3	1	6	7
poldmE8-80-2	7	5	12
poldmE8-80-3	0	5	5
poldmE8-100-1	7	3	10
**poldmF11-1-1**	**0**	**0**	**0**
poldmF11-20-1	5	7	12
poldmF11-20-2	5	7	12
poldmF11-20-3	2	8	10
poldmF11-40-1	5	10	15
poldmF11-40-2	9	7	16
poldmF11-60-1	3	12	15
poldmF11-60-2	11	8	19
poldmF11-60-3	6	9	15
poldmF11-80-1	9	6	15
poldmF11-80-2	9	7	16
poldmF11-80-3	13	9	22
poldmF11-100-1	3	10	13
poldmF11-100-2	13	9	22
poldmF11-100-3	7	7	14
**poldmH11-1-1**	**0**	**0**	**0**
poldmH11-20-1	6	15	21
poldmH11-20-2	7	11	18
poldmH11-20-3	3	10	13
poldmH11-40-1	4	12	16
poldmH11-40-2	4	9	13
poldmH11-40-3	8	8	16
poldmH11-60-1	7	9	16
poldmH11-60-2	4	9	13
poldmH11-60-3	8	6	14
poldmH11-80-1	5	8	13
poldmH11-100-1	8	14	22

Supplementary Table 3: Identical and nearby mutations arising in different lines.

Gene ID	Gene Name	Mutation Type	Position	Nucleotide	Lines Containing Mutations	Additional Nearby (<50bp) Mutations	Distance to Nearby Mutation (bp)
PfDd2_020016900	conserved Plasmodium protein, unknown function	Intergenic (upstream)	471545	c.-4875C>T	Dd2, Dd2Polδ-H11	YES	2
PfDd2_060034400	SET domain protein, putative	Intergenic (upstream)	1189896	c.-643T>C	Dd2Polδ-F11, Dd2Polδ-H11	NO	
PfDd2_070021100	calcium-dependent protein kinase 4	Intergenic (upstream)	715218	c.-686T>A	Dd2, Dd2Polδ-E8, Dd2Polδ-H11	NO	
PfDd2_070029500	DNA mismatch repair protein PMS1, putative	Intergenic (downstream)	1067187	c.*192A>T	Dd2, Dd2Polδ-F11	NO	
PfDd2_090020800	cytochrome c oxidase subunit ApiCOX30, putative	Intergenic (upstream)	659167	c.-2354T>A	Dd2, Dd2Polδ-H11	NO	
PfDd2_110020500	tyrosine--tRNA ligase	Intergenic (upstream)	580315	c.-2929G>A	Dd2Polδ-F11, Dd2Polδ-H11	YES (2)	3, 8
PfDd2_110036900	conserved Plasmodium protein, unknown function	Intergenic (upstream)	1214701	c.-40T>A	Dd2, Dd2Polδ-E8, Dd2Polδ-H11	YES	3
			1214704	c.-43T>C	Dd2, Dd2Polδ-E8, Dd2Polδ-F11, Dd2Polδ-H11		
PfDd2_120023100	triose or hexose phosphate/phosphate translocator, putative	Intergenic (upstream)	701420	c.-1747A>G	Dd2Polδ-E8, Dd2Polδ-H11	NO	
PfDd2_120051300	delta-aminolevulinic acid synthetase	Intergenic (upstream)	1916041	c.-1763A>T	Dd2Polδ-E8, Dd2Polδ-H11	YES	14
PfDd2_130031000	phosphoribosylpyrophosphat e synthetase	Intergenic (upstream)	1060382	c.-2587T>A	Dd2Polδ-F11, Dd2Polδ-H11	NO	
PfDd2_140050000	RNA-binding protein, putative	Intergenic (downstream)	1854987	c.*966A>C	Dd2Polδ-E8, Dd2Polδ-F11	YES	7
			1854994	c.*973T>C	Dd2, Dd2Polδ-E8, Dd2Polδ-F11, Dd2Polδ-H11		
PfDd2_140067300	conserved Plasmodium protein, unknown function	Intergenic (upstream)	2544236	c.-1417T>C	Dd2, Dd2Polδ-F11	NO	

Supplementary Table 4. Summary of unsuccessful MMV665794 selections using wild type 3D7 and Dd2 strains.

Parasite line	Selection method	Outcome
3D7-WT	Constant pressure at $3xIC_{50}$ for 45 days	No parasites reappeared
3D7-WT	Constant pressure at $1.5xIC_{50}$ for 45 days	No parasites reappeared
Dd2-WT	Constant pressure at $3xIC_{50}$ for 45 days	No parasites reappeared
Dd2-WT	Ramp up/on-off starting at $1xIC_{50}$ or $3xIC_{50}$ for 45 days	No parasites reappeared

Supplementary Table 5. Single guide RNAs and sequencing primers for verifying CRISPR plasmid constructs and CRISPR-edited parasites.

Primer name	Sequences (5'-3')
SgRNA1 DNA pol δ D308A-E310A-F	TATTGAAGGATAAAATTCTTAACTT
SgRNA1 DNA pol δ D308A-E310A-R	AAACAAGTTAAGAATTTTATCCTTC
SgRNA2 DNA pol δ D308A-E310A-F	TATTGTGTATAAAATTAGACGGTAA
SgRNA2 DNA pol δ D308A-E310A-R	AAACTTACCGTCTAATTTTATACAC
p345-donor template-F for CRISPR plasmid sequencing	GAGGTACCGAGCTCGAATTC
p346-donor template-R for CRISPR plasmid sequencing	CGAAAAGTGCCACCTGACGTC
p35-gRNA sequencing-F for CRISPR plasmid sequencing	AAGCACCGACTCGGTGCCAC
p1629-gRNA sequencing-R for CRISPR plasmid sequencing	GTGTAGTTAATTCATCAAATAGCATGCC
p1495-PCR-CRISPR-edited-F for pol δ D308A-E310A	GTTGAGAAACCTGATGATTTTGATAATG
p1496-PCR-CRISPR-edited-R for pol δ D308A-E310A	CCATCTAACTTAATACATGCTATAGC
p1497-PCR-non-CRISPR-edited-R for pol δ D308A-E310A	CCGTCTAATTTTATACATTCAATATCAAAG
p1510-sequencing-CRISPR-edited-R for pol δ D308A-E310A	GCGTACCAAACTGTTTAGATGAGAAAC
P1511-sequencing-CRISPR-edited-F for pol δ D308A-E310A	GATCATAAGATTACAGGTTCATCCTGG
SgRNA1 QRP1 G1612V-F	TATTCATATTATAAGTCACTCATG
SgRNA1 QRP1 G1612V-R	AAACCATGAGTGACTTATAATATG
SgRNA2 QRP1 G1612V-F	TATTAATTGTCCTCTACACAACTT
SgRNA2 QRP1 G1612V-R	AAACAAGTTGTGTAGAGGACAATT
SgRNA1 QRP1 D1863Y-F	TATTGTTTCTGAATATGTTAAAGCT
SgRNA1 QRP1 D1863Y-R	AAACAGCTTTAACATATTCAGAAAC
SgRNA2 QRP1 D1863Y-F	TATTCTTTAACATATTCAGAAACA
SgRNA2 QRP1 D1863Y-R	AAACTGTTTCTGAATATGTTAAAG
Genotyping primer-F for G1612V/G1612 silent control	CGATAGGTAATAAAATGAGGAATTTG
Genotyping primer-R for G1612V/G1612 silent control	TAGTTAAGTTGTTACTCGAATGC
Genotyping primer-F for D1863Y/D1863 silent control	CATTCGAGTAACAACTTAACTAATAG
Genotyping primer-R for D1863Y/D1863 silent control	CACTATCACAAACTTTATACTGCT

Supplementary Table 6. The unique and duplicate reads from WGS and % of genome having read depth > 10 and > 5.

Name	Unique Reads	Duplicate Reads	% read depth >10	% read depth >5
Dd2-1-1	14769170	3040808	99.51	99.97
Dd2-100-1	5952540	443763	93.77	98.15
Dd2-20-1	15066292	3025525	99.45	99.93
Dd2-20-2	18609611	5016444	99.06	99.85
Dd2-40-1	8481909	999102	96.19	99.14
Dd2-40-2	6611272	561438	90.98	96.94
Dd2-40-3	7287968	664877	96.24	99.05
Dd2-60-1	7565158	792763	94.56	98.42
Dd2-60-2	7069327	639142	94.45	98.59
Dd2-60-3	7542181	738795	94.66	98.55
Dd2-80-1	7264230	773332	92.46	97.59
Dd2-80-2	7138064	716103	90.91	96.79
Dd2-80-3	6463084	522766	92.23	97.64
poldmE8-1-1	5893083	607360	88.25	95.36
poldmE8-100-1	7800072	760535	98.14	99.62
poldmE8-20-2	7730155	823145	94.99	98.57
poldmE8-20-3	7038999	584647	96.26	99.11
poldmE8-40-1	6884270	647710	92.14	97.51
poldmE8-40-2	6473538	523251	92.57	97.63
poldmE8-40-3	8126877	934113	93.89	98.32
poldmE8-60-1	6006496	448769	92.93	97.91
poldmE8-60-2	6946042	640212	94.67	98.62
poldmE8-60-3	6130348	541556	90.71	96.87
poldmE8-80-1	5835502	412587	92.51	97.56
poldmE8-80-2	6587823	569450	93.74	98.11
poldmE8-80-3	5946965	475073	92.20	97.55
poldmF11-1-1	6057568	528834	88.28	95.65
poldmF11-100-1	8319731	1161991	95.26	98.86
poldmF11-100-2	6648267	551164	96.02	99.15
poldmF11-100-3	7669947	836794	95.24	98.70
poldmF11-20-1	5791224	456442	89.21	95.92
poldmF11-20-2	6964323	650971	94.51	98.44
poldmF11-20-3	6318404	508672	94.27	98.44
poldmF11-40-1	8427775	1058351	95.83	98.99
poldmF11-40-2	7498668	658184	95.81	98.85
poldmF11-60-1	6292506	475844	95.46	98.82
poldmF11-60-2	6182210	501781	92.66	97.82
poldmF11-60-3	7532574	911423	92.20	97.59
poldmF11-80-1	6659100	674351	89.43	96.02
poldmF11-80-2	7318953	718796	94.61	98.44
poldmF11-80-3	7160713	632422	94.92	98.59
poldmH11-1-1	6420885	538333	92.05	97.42
poldmH11-100-1	6625345	621906	93.61	98.06
poldmH11-20-1	7736958	828564	94.72	98.50
poldmH11-20-2	6048678	497831	89.92	96.62
poldmH11-20-3	6914208	657475	93.37	97.94
poldmH11-40-1	6707343	563439	94.73	98.63

Name	Unique Reads	Duplicate Reads	% read depth >10	% read depth >5
poldmH11-40-2	7023534	646913	94.57	98.39
poldmH11-40-3	6702375	612303	91.32	97.10
poldmH11-60-1	9004269	1079027	96.28	98.99
poldmH11-60-2	6633479	570458	93.89	98.09
poldmH11-60-3	7124799	662045	95.13	98.67
poldmH11-80-1	6170512	538337	90.39	96.58
poldmH11-nodrug-F1	7686874	828749	95.52	98.90
poldmH11-nodrug-F2	6745022	622075	94.28	98.53
poldmH11-nodrug-F3	6424501	557473	95.66	98.92
poldmH11-MMV665794-clone1	7724812	927771	95.27	98.86
poldmH11-MMV665794-clone2	5886563	464846	90.49	96.91
poldmH11-MMV665794-clone3	6805012	686455	93.56	98.08
poldmH11-MMV665794-clone4	6424965	579556	91.97	97.43
poldmH11-MMV665794-clone5	8110864	1005500	94.41	98.57
poldmH11-KAE609-clone1	7238083	791409	93.81	98.07
poldmH11-KAE609-clone2	6595094	709986	89.44	96.41
poldmH11-KAE609-clone3	6482554	623836	90.98	97.12
