# Supplementary material for: Generation of a mutator parasite to drive resistome discovery in Plasmodium falciparum
Source: Nat Commun. 2023 May 27;14:3059. doi: 10.1038/s41467-023-38774-1 (PMC10224993; doi:10.1038/s41467-023-38774-1)
Supplement: Supplementary file 3 — Description of additional supplementary files [file 41467_2023_38774_MOESM3_ESM.docx]

**Title:** Supplementary Data 1

**Description:** List of SNVs in Dd2-WT and Dd2-Polδ occurring during the mutation accumulation assay identified by whole genome sequencing.

**Title:** Supplementary Data 2

**Description:** Mutation rate calculation.

**Title:** Supplementary Data 3

**Description:** SNVs in drug-selected lines in the Dd2-Polδ-H11 identified by whole genome sequencing.

**Title:** Supplementary Data 4

**Description:** CNV analysis: Denoised Log_2_ copy ratios for MMV665794-selected clones.
