# Supplementary material for: Generation of a mutator parasite to drive resistome discovery in Plasmodium falciparum
Source: Nat Commun. 2023 May 27;14:3059. doi: 10.1038/s41467-023-38774-1 (PMC10224993; doi:10.1038/s41467-023-38774-1)
Supplement: Supplementary file 8 — Reporting Summary [file 41467_2023_38774_MOESM8_ESM.pdf]

nature portfolio

Corresponding author(s): Marcus Lee

Last updated by author(s): 30/4/2023

# Reporting Summary

Nature Portfolio wishes to improve the reproducibility of the work that we publish. This form provides structure for consistency and transparency in reporting. For further information on Nature Portfolio policies, see our Editorial Policies and the Editorial Policy Checklist.

## Statistics

For all statistical analyses, confirm that the following items are present in the figure legend, table legend, main text, or Methods section.

| n/a | Confirmed | |
|---|---|---|
| ☐ | ☒ | The exact sample size ($n$) for each experimental group/condition, given as a discrete number and unit of measurement |
| ☐ | ☒ | A statement on whether measurements were taken from distinct samples or whether the same sample was measured repeatedly |
| ☐ | ☒ | The statistical test(s) used AND whether they are one- or two-sided<br>*Only common tests should be described solely by name; describe more complex techniques in the Methods section.* |
| ☒ | ☐ | A description of all covariates tested |
| ☐ | ☒ | A description of any assumptions or corrections, such as tests of normality and adjustment for multiple comparisons |
| ☐ | ☒ | A full description of the statistical parameters including central tendency (e.g. means) or other basic estimates (e.g. regression coefficient) AND variation (e.g. standard deviation) or associated estimates of uncertainty (e.g. confidence intervals) |
| ☐ | ☒ | For null hypothesis testing, the test statistic (e.g. $F$, $t$, $r$) with confidence intervals, effect sizes, degrees of freedom and $P$ value noted<br>*Give P values as exact values whenever suitable.* |
| ☒ | ☐ | For Bayesian analysis, information on the choice of priors and Markov chain Monte Carlo settings |
| ☒ | ☐ | For hierarchical and complex designs, identification of the appropriate level for tests and full reporting of outcomes |
| ☒ | ☐ | Estimates of effect sizes (e.g. Cohen's $d$, Pearson's $r$), indicating how they were calculated |

*Our web collection on statistics for biologists contains articles on many of the points above.*

## Software and code

Policy information about availability of computer code

| | |
|---|---|
| Data collection | Flow cytometry on BeckmanCoulter Cytoflex S running CytExpert v 2.4; DNA quantitation on Qubit 2.0; Drug susceptibility assays on BMG FluorStar Omega v5.11; Cell counts on Cellometer Auto 1000 (Nexcelom) |
| Data analysis | GraphPad Prism versions 8-9; R v4.1.3; Microsoft Excel v16; PyMOL 2.5.2; CytExpert v2.4; FlowJo v10; Burrows-Wheeler Aligner (bwa/0.7.17); GATK MarkDuplicates (picard/2.22.2--0); GATK HaplotypeCaller (gatk/4.1.4.1); snpEff (vX); |

For manuscripts utilizing custom algorithms or software that are central to the research but not yet described in published literature, software must be made available to editors and reviewers. We strongly encourage code deposition in a community repository (e.g. GitHub). See the Nature Portfolio guidelines for submitting code & software for further information.

## Data

Policy information about availability of data

All manuscripts must include a data availability statement. This statement should provide the following information, where applicable:

- Accession codes, unique identifiers, or web links for publicly available datasets
- A description of any restrictions on data availability
- For clinical datasets or third party data, please ensure that the statement adheres to our policy

The data underlying this article are available within the supplementary material files. All associated sequence data are available at the NCBI Sequence Read Archive under accession code ERP110649 (BioProject: PRJEB2844). Library names DN581642P:A7, D7, E7 and DN573783H:A5-12, B5-B12, C5-C12, D5-D8, D10-D12, E5-E12, F5-F12, G6-G7, G9-G12, H4-H12. Reference genomes for Dd2 and 3D7 and gene annotation data are available on PlasmoDB (Dd2 at https://plasmodb.org/

common/downloads/release-44/PfalciparumDd2/; 3D7 at https://plasmodb.org/common/downloads/release-44/Pfalciparum3D7/)

## Human research participants

Policy information about studies involving human research participants and Sex and Gender in Research.

| | |
|---|---|
| Reporting on sex and gender | Red blood cells (RBCs) were used for culture of Plasmodium falciparum parasites used in this study. RBCs were obtained from anonymous donors from the National Health Services Blood and Transplant (NHSBT, UK) or Red Cross (Madrid, Spain). |
| Population characteristics | RBCs were obtained from anonymous healthy donors. |
| Recruitment | Not applicable, as no specific recruitment was performed. RBCs were collected from anonymous donors to the health services listed above. |
| Ethics oversight | The use of RBCs was performed in accordance with relevant guidelines and regulations, with approval from the NHS Cambridgeshire Research Ethics Committee and the Wellcome Sanger Institute Human Materials and Data Management Committee for the experiments performed in the UK. RBCs were sourced ethically and their research use was in accord with the terms of the informed consents under an IRB/EC approved protocol for experiments done in Spain |

Note that full information on the approval of the study protocol must also be provided in the manuscript.

# Field-specific reporting

Please select the one below that is the best fit for your research. If you are not sure, read the appropriate sections before making your selection.

☒ Life sciences ☐ Behavioural & social sciences ☐ Ecological, evolutionary & environmental sciences

For a reference copy of the document with all sections, see nature.com/documents/nr-reporting-summary-flat.pdf

# Life sciences study design

All studies must disclose on these points even when the disclosure is negative.

| | |
|---|---|
| Sample size | Sample size was not predetermined. Samples sizes of three to six independent experiments were performed for all drug assays, and two independent experiments for the competitive fitness experiment. In vitro evolution experiments were performed on three independent selections. Sample sizes were based on previously published work from authors in this study, e.g. PMID: 36927839 (Bopp et al., 2023, Nat Comm); PMID: 28262680 (Sonoiki et al., 2017, Nat Comm), PMID: 36888694 (Istvan et al., 2023, Science). |
| Data exclusions | One biological replicate of one drug assay was excluded, as one of the two technical replicates failed to receive drug. |
| Replication | All experiments were performed with multiple replicates (three - six biological replicates with technical duplicates for drug assays; two biological replicates with technical triplicates for competitive fitness), with replicates noted within the manuscript. All results were replicated successfully. |
| Randomization | Selection of clonal lines for expansion and whole genome sequencing to determine mutation rates of wild type and DNA-Pol lines were chosen randomly. Randomization was not applied to other experiments, as different parasite cultures were prepared and tested in the same manner. |
| Blinding | Investigators were not blinded in this study, as experiments were based on objective and quantitative measurements that would minimize investigator bias in interpretation. |

# Reporting for specific materials, systems and methods

We require information from authors about some types of materials, experimental systems and methods used in many studies. Here, indicate whether each material, system or method listed is relevant to your study. If you are not sure if a list item applies to your research, read the appropriate section before selecting a response.

## Materials & experimental systems

| n/a | Involved in the study |
|---|---|
| ☒ | ☐ Antibodies |
| ☐ | ☒ Eukaryotic cell lines |
| ☒ | ☐ Palaeontology and archaeology |
| ☒ | ☐ Animals and other organisms |
| ☒ | ☐ Clinical data |
| ☒ | ☐ Dual use research of concern |

## Methods

| n/a | Involved in the study |
|---|---|
| ☒ | ☐ ChIP-seq |
| ☐ | ☒ Flow cytometry |
| ☒ | ☐ MRI-based neuroimaging |

# Eukaryotic cell lines

Policy information about cell lines and Sex and Gender in Research

| | |
|---|---|
| Cell line source(s) | The original parental Dd2 strain was obtained from Dr. David Fidock, Columbia University Medical Center. Red blood cells were obtained from the NHSBT, UK or were purchased from Interstate Blood Bank, USA. |
| Authentication | Parasite lines were authenticated by whole genome sequencing. |
| Mycoplasma contamination | The Dd2 and DNA-Pol lines used for this study were tested and shown to be negative. |
| Commonly misidentified lines (See ICLAC register) | No commonly misidentified lines were used. |

# Flow Cytometry

## Plots

Confirm that:

☒ The axis labels state the marker and fluorochrome used (e.g. CD4-FITC).

☒ The axis scales are clearly visible. Include numbers along axes only for bottom left plot of group (a 'group' is an analysis of identical markers).

☒ All plots are contour plots with outliers or pseudocolor plots.

☒ A numerical value for number of cells or percentage (with statistics) is provided.

## Methodology

| | |
|---|---|
| Sample preparation | Samples were prepared in a 96-well round-bottom plate (Costar) by taking 4 uL of culture into 200 uL PBS containing 100 nM of Mitotracker Deep Red FM. The plate was incubated at 37C for 15 minutes before analysis on the flow cytometer. The gates were set up for the FITC (gain 5 or 10) and APC (gain 3 or 5) channels for GFP and Mitotracker Deep Red FM signals, respectively. |
| Instrument | BeckmanCoulter Cytoflex S |
| Software | CytExpert v2.4; FlowJo v10 |
| Cell population abundance | Cell population of infected red blood cells was set at 20,000 based on staining with Mitotracker Deep Red. |
| Gating strategy | Gating was based initially on the uninfected RBC sample population stained with Mitotracker Deep Red to define non-parasitized cells. The query line (GFP negative) and reference line (GFP positive) were also stained with Mitotracker Deep Red, and examined in the FITC vs APC channels, with boundaries defined by running samples with either non-fluorescent parasites (Dd2 or DNA-Pol) or fluorescent parasites (Dd2-GFP) only. |

☒ Tick this box to confirm that a figure exemplifying the gating strategy is provided in the Supplementary Information.
